# Enhancing host-pathogen phenotyping dynamics: early detection of tomato bacterial diseases using hyperspectral point measurement and predictive modeling

**DOI:** 10.3389/fpls.2023.1242201

**Published:** 2023-08-16

**Authors:** Mafalda Reis Pereira, Filipe Neves dos Santos, Fernando Tavares, Mário Cunha

**Affiliations:** ^1^ Faculdade de Ciências da Universidade do Porto (FCUP), Rua do Campo Alegre, Porto, Portugal; ^2^ Institute for Systems and Computer Engineering, Technology and Science (INESC TEC), Campus da Faculdade de Engenharia da Universidade do Porto, Rua Dr. Roberto Frias, Porto, Portugal; ^3^ CIBIO, Centro de Investigação em Biodiversidade e Recursos Genéticos, InBIO Laboratório Associado, Campus de Vairão, Universidade do Porto, Vairão, Portugal; ^4^ BIOPOLIS Program in Genomics, Biodiversity and Land Planning, CIBIO, Campus de Vairão, Vairão, Portugal

**Keywords:** plant disease diagnosis, early diagnosis, proximal sensing, hyperspectral spectroscopy, point of measurement, applied aredictive modeling, linear discriminant analysis, machine learning

## Abstract

Early diagnosis of plant diseases is needed to promote sustainable plant protection strategies. Applied predictive modeling over hyperspectral spectroscopy (HS) data can be an effective, fast, cost-effective approach for improving plant disease diagnosis. This study aimed to investigate the potential of HS point-of-measurement (POM) data for in-situ, non-destructive diagnosis of tomato bacterial speck caused by *Pseudomonas syringae* pv. *tomato* (Pst), and bacterial spot, caused by *Xanthomonas euvesicatoria* (Xeu), on leaves (cv. cherry). Bacterial artificial infection was performed on tomato plants at the same phenological stage. A sensing system composed by a hyperspectral spectrometer, a transmission optical fiber bundle with a slitted probe and a white light source were used for spectral data acquisition, allowing the assessment of 3478 spectral points. An applied predictive classification model was developed, consisting of a normalizing pre-processing strategy allied with a Linear Discriminant Analysis (LDA) for reducing data dimensionality and a supervised machine learning algorithm (Support Vector Machine – SVM) for the classification task. The predicted model achieved classification accuracies of 100% and 74% for Pst and Xeu test set assessments, respectively, before symptom appearance. Model predictions were coherent with host-pathogen interactions mentioned in the literature (e.g., changes in photosynthetic pigment levels, production of bacterial-specific molecules, and activation of plants’ defense mechanisms). Furthermore, these results were coherent with visual phenotyping inspection and PCR results. The reported outcomes support the application of spectral point measurements acquired *in-vivo* for plant disease diagnosis, aiming for more precise and eco-friendly phytosanitary approaches.

## Graphical Abstract

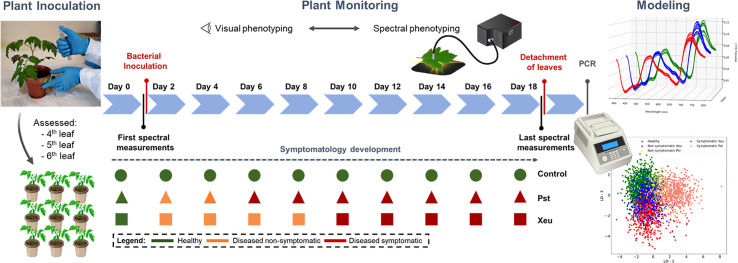

## 1 Introduction

The tomato (*Solanum lycopersicum* L.) crop holds great importance worldwide due to its significant impact on agriculture, the economy, and human nutrition. This globally cultivated vegetable crop is very sensitive to diseases leading to dramatic yield and economic losses (Blancard, 2012). Bacterial diseases of tomato plants caused by the Gram-negative bacteria *Pseudomonas syringae* pv. *tomato* (Pst, bacterial speck) and *Xanthomonas euvesicatoria* (Xeu) formerly known as *Xanthomonas campestris* pv. *vesicatoria*, bacterial spot, are two important etiological agents responsible for several plant outbreaks and considerable losses in tomato production worldwide. These two diseases are responsible for severe alterations in the host physiology, biochemistry, and structural composition, causing plant phenotype modifications (e.g., reduction of the photosynthetic capacity of diseased foliage, defoliation, flower abortion, and fruit lesions, among others). Ultimately, they result in yield reductions due to the damage caused to plants and fruits, which makes them unsuitable for the fresh market or processing. Control measures for these two crop diseases may be ineffective, especially when the bacteria are well-established in a production site (medium to late stage of the disease infection process. Phytosanitary products, such as copper and antibiotics (Alves et al., 2023), can be applied to mitigate the negative effects of the disease. Nevertheless, this approach can lead to bacteria tolerance to phytosanitary compounds (Blancard, 2012), and conduct to considerable damage to the environment and food security due to non-targeted applications of these products (Zhang et al., 2020).

Nowadays, bacterial diseases are diagnosed essentially through scouting and ‘wet lab’ -based approaches. The first requires a careful and detailed inspection of crop fields (usually visual) by specialized trained observers. They must detect and identify diseased plants based on modifications to the characteristic phenotype of the crop, and the presence of disease symptoms (Parker et al., 1995). Thus, it is subjective, error-prone (as symptoms alone are not entirely disease-specific, and can be promoted by other biotic and abiotic stresses), labor-intensive, time-consuming, and expensive (Mahlein, 2016). In turn, laboratory-based techniques consist of serological and molecular assays, frequently applied due to their sensitivity, accuracy, and effectiveness. The most widespread lab methods include Enzyme-Linked Immunosorbent Assay (ELISA) and Polymerase Chain Reaction (PCR) methods. They involve comprehensive sampling procedures, which require several hours to be completed, and destructive sample preparation, precluding the accompaniment of disease development nor its field mapping to support precision agriculture systems (e.g. Site-Specific Management) (Fang and Ramasamy, 2015; Martinelli et al., 2015). Nevertheless, laboratory-based approaches lack appropriate high throughput and speed for supporting real-time agronomic precision decisions in-field since they were developed to verify the presence of pathogens. They also still have some diagnostic constraints, mostly in the non-symptomatic and early disease infection stages, related to the irregular spread of bacteria inside plants (Fang and Ramasamy, 2015; Martinelli et al., 2015).

Hyperspectral spectroscopy (HS) is one innovative approach that has been studied and successfully applied to assess different plant(host)-pathogen interactions in a fast, sensitive, standardized cost-effective, high-throughput, and non-invasive way (Golhani et al., 2018). Through spectral measurements in the visible (VIS, 400-700 nm) and infrared (IR, 800-2500 nm) regions, HS showed the capability of effectively assessing a wide variety of plant structural, chemical, biophysical, and metabolic traits in living tissues (Thenkabail et al., 2000; Delalieux et al., 2007). Changes in the typical spectral phenotype of a crop may indicate deviations in its health status, leading to an indirect method of diagnosing diseases. Plant-pathogen interactions shift plant metabolism and tissue composition, resulting in detectable variations in the plant’s optical behavior. In brief, these dynamics typically promote modifications in the VIS spectra of plants, due to changes in pigments’ concentration and physiological processes. Furthermore, variations in the IR region may also occur and are essentially linked to leaf water levels, chemical compounds (namely lignin’s and proteins content), structural elements, and internal scattering processes (Thenkabail et al., 2014; Tosin et al., 2022).

Different types of pathogens, such as pests (Herrmann et al., 2017; Zhang et al., 2017), fungi (Yu et al., 2018; Skoneczny et al., 2020), bacteria (Bagheri et al., 2018), and viruses (Morellos et al., 2020) affecting different crops have already been detected using the HS technique, mostly in symptomatic stages. Thus, this spectral phenotyping technique constitutes an interesting diagnosis method, allowing the distinction between the spectral signature of healthy and disease tissues, as well as between the spectral signature of diseased tissues infected with different pathogens.HS holds great potential for early disease diagnosis, i.e., when plants are diseased but still don’t manifest any visual symptoms of the infection (Gold et al., 2020a; Reis-Pereira et al., 2022). However, the use of this approach for non-symptomatic plant disease diagnosis remains largely unexplored. Understanding host-pathogen specific interactions and overcoming technical challenges related to the biophysical status of infected plants, organ of the plant assessed, sensing technology, data processing, and modeling approaches is essential for the effective application of HS *in vivo* crop disease diagnosis (Mahlein et al., 2018). Addressing these challenges is crucial for real-time monitoring of disease progression.

The most used sensing devices for plant disease detection are non-imaging (e.g., point-of-measurement, POM) and imaging sensors. In POM sensing, light usually enters the leaf, and undergoes internal reflections conditioned by tissue structures and composition status. Thus, this technique can indirectly infer certain internal tissue characteristics affected by the host-pathogen interaction. POM sensors are typically designed to measure specific parameters without being significantly affected by factors like lighting conditions or surface textures. This reduces the potential for external interference and ensures more accurate and consistent measurements. This allied with their higher spectral resolution, cost-effectiveness, compactness, and reduced data processing requirements, makes them an attractive option for plant studies (Martins et al., 2022).

Spectral information provided by HS is extremely valuable, nonetheless, in biological tissues, it is super-imposed in the recorded spectra at different scales of interference (Barroso et al., 2022; Tosin et al., 2022). Moreover, HS data can present substantial amounts of redundant information in contiguous wavelengths, and just some specific spectral features might be relevant to predict and classify diseased tissues (Caicedo et al., 2014; Rivera et al., 2014). Applied predictive classification modeling strategies can be developed to study spectral data and extract useful information. Diverse approaches of data correction and pre-processing (e.g., data scaling and normalization) can be computed to reduce undesired spectral effects, such as ‘noise’ and scattering effects. Additionally, modeling strategies, as well as feature selection (FS), feature extraction and dimensionality reduction techniques (DR), can be useful for determining the wavelength features which have more influence in disease discrimination (Mahlein et al., 2010; Ahmadi et al., 2017). In plant disease research, different predictive approaches using HS data have been explored to classify tissues affected by biotic stress, considering all the spectral features or only specific variables, designated by FS or DR techniques (Gold et al., 2020b; Meng et al., 2020). Nevertheless, there is a lack of standardized protocols for acquiring hyperspectral data from tomato leaves. Different studies employ various acquisition setups, lighting conditions, and preprocessing techniques, making comparing and integrating findings challenging.

This work addresses the main technological challenges for efficiently applying hyperspectral technologies in phenotyping to diagnose plant diseases. Conducting analysis for healthy and bacterial inoculated plants over time, this study aims i) to compare visual phenotyping against spectral phenotyping based on the hyperspectral point-of-measurement (HS-POM) for healthy and diseased tomato leaflets, ii) to evaluate the HS-POM ability to accurately classify samples at various stages of disease development, including those without any visible symptoms and iii) distinguish the etiological agents of distinct tomato bacterial diseases. The specific goals include developing an applied predictive modeling strategy (combining data pre-processing, dimensionality reduction, and a supervised machine learning algorithm) for tomato bacterial disease classification and establishing causal relationships between plant health status, specific spectra characteristics, and the physiological changes that occur during infection dynamics to advance theoretical knowledge and provide a foundation for further research.

## 2 Materials and methods

### 2.1 Bacterial inoculation and plant growth

#### 2.1.1 Inoculation on tomato leaflets

**Figure 1:**
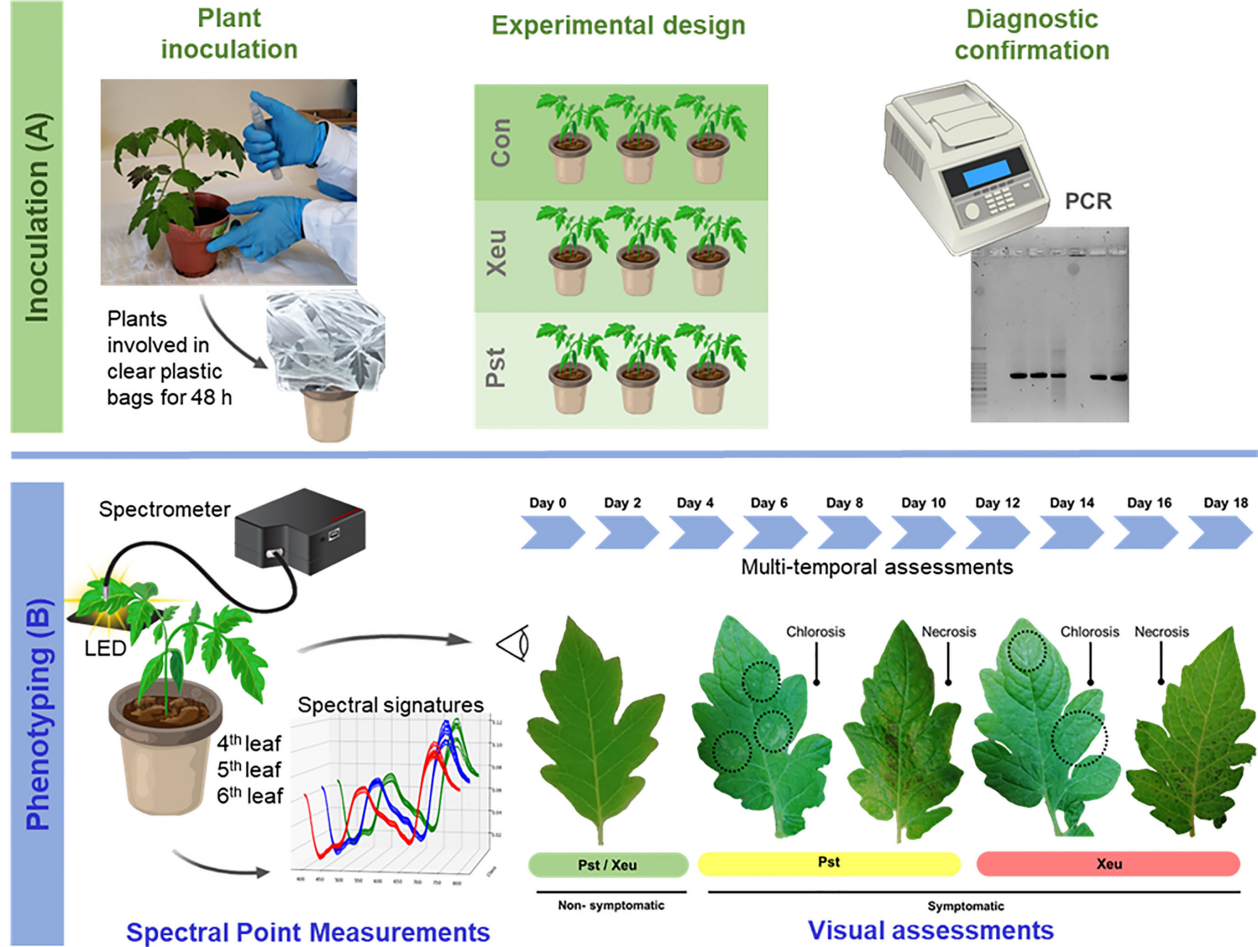
Experimental setup of the bacterial inoculation assay performed on tomato leaves **(A)**, and visual and spectral assessments (of the 4^th^, 5^th^, and 6^th^ leaves) made in a dark room **(B)**. Spectral measurements were performed on the adaxial side of leaflets, using a spectrometer combined with an optical fiber bundle with a reflection probe. A white LED was placed beneath each leaflet. Both visual and spectral assessments were made 18 Days After Inoculation (DAI), collecting leaflets’ spectral signatures and registering modifications in their phenotype (e.g., the appearance of the first symptoms, both chlorosis and necrosis).

Tomato (*Solanum lycopersicum* L.) plants of the cultivar Cherry were grown in 200 mL pots containing a commercial potting substrate, in a walk-in plant growth chamber under controlled conditions (25-27 °C, humidity of approximately 60%, photoperiod of 12/12 h and light intensity 30W). Plants were divided into three groups of three plants each (nine plants in total), being a) one group of plants inoculated with *Pseudomonas syringae* pv. *tomato* DC 3000 (Pst) bacteria, b) a second group of plants inoculated with *Xanthomonas euvesicatoria* LMG 905 (Xeu) bacteria, and c) a third group of plants was treated with sterile distilled water only (Control group) (**Figure 1**). Plants were physically separated to avoid cross-contamination.

Plants were inoculated in the laboratory, at the growth stage of 5-6 fully expanded leaves, by spraying until they became fully wet, and run-off occurred. The bacterial suspensions used for these inoculation assays consisted of 1 x 10^8^ cells/mL. They were prepared from 48-h-old cultures of Pst grown in KB medium (peptone, 20.0g; K_2_HPO^4^, 1.5g; MgSO^4^, 1.5g; glycerol, 10 mL; agar, 15g; distilled water up to 1.0 liter), and of Xeu cultures grown in YDC medium (yeast extract, 10.0g; dextrose, 20.0g; CaCO_3_, 20.0g; agar, 15.0g; distilled water up to 1.0 liter). The inoculated plants were then covered with transparent polythene bags for 48 h to increase the relative humidity that fosters bacterial entry into plant tissues through natural openings such as stomata (Lamichhane, 2015). Plants were monitored daily for symptom development for 18 days (**Figure 1**).

During the inoculation period, to verify if the bacteria cultures used in these inoculation tests were viable, 20 μL of Pst solution and 20 μL of Xeu solution were cultured in Petri dishes containing KB and YDC media, respectively. After 48 h was possible to observe the bacteria growth in both nutrient media, proving that bacteria were viable at the moment of inoculation.

#### 2.1.2 Bacterial isolation from diseased leaflets

After the last spectral measurement, sample preparation for bacterial isolation was performed for all the leaflets. Leaflets were excised from plants using a sterile scalpel (Fernandes et al., 2017). Bacterial isolation was carried out as defined by Fernandes et al. (2017; 2021). Briefly, each sample of excised leaflet tissue was disinfected by immersion in 70% ethanol followed by washing with sterile distilled water (SDW), and then macerated with SDW in extraction bags. The suspensions obtained, and corresponding dilutions, were streaked on KB (samples inoculated with Pst bacteria), and on YDC medium (samples infected with Xeu pathogen). Characteristic colonies from these two bacteria species (milky white colonies in the case of Pst, and mucoid yellow colonies in the case of Xeu) were selected for growth on fresh nutrient agar medium to ensure purity.

Pst characteristic symptoms resemble small greasy dark stains (circular or slightly angular), that become brown to black, and appear randomly on the leaflets (often on the youngest or the ones located at the edge of the canopy plant). These lesions may typically show a yellow halo of various sizes. They are about 2–3 mm and can develop and coalesce (especially in the presence of moisture), affecting large areas of the leaf, that may later become necrotic and desiccate (Blancard, 2012). In turn, Xeu characteristic symptoms comprise small, circle, or slightly angular, translucent, and water-soaked lesions, which turn brown with time. They appear randomly in leaflets, and eventually become necrotic spots, with light gray centers and dark margins, which also can become surrounded by a yellow hallow with time. Smaller lesions can coalesce into each other forming larger injuries, whose diameter can range from 2 to 3 mm. In severe cases, tissues in the center of a lesion become dry and fall out, leading to “shot-hole” symptoms (Ritchie, 2000; Blancard, 2012).

#### 2.1.3 Colony PCR protocol

A colony PCR was performed to validate the presence of both bacteria species on tomato leaflets isolates. PST2 (Vieira et al., 2007) and XV14 (Albuquerque et al., 2012) were the chosen markers, for Pst and Xeu, respectively, with amplicon lengths of 200, and 713 bp, correspondingly. A 20 µL PCR reaction mix consisted of 1 × DreamTaq Buffer (ThermoFisher Scientific, Waltham, MA, USA), 0.2 mM of each deoxynucleotide triphosphate (dNTP) (Grisp, Porto, Portugal), 0.2 mM of each forward and reverse primers, 1 U of DreamTaq DNA Polymerase (ThermoFisher Scientific, Waltham, MA, USA) and 10 µL of DNA isolate solution. Sterile distilled water was used as the negative control. PCR cycling parameters were defined as stated by Vieira et al. (2007) for Pst, and Albuquerque et al. (2012) for Xeu. PCR products were then separated by electrophoresis on a 0.8% agarose gel (1 × TAE buffer) and visualized using Xpert Green DNA stain (Grisp, Porto, Portugal) with a Molecular Imager Gel Doc XR+ System (Bio-Rad, Hercules, CA, USA).

### 2.2 Spectral measurements *in vivo* tissue

#### 2.2.1 Experimental setup for plant spectral acquisition

**Figure 1** presents the main procedures for spectral measurements in the experimental setup. Hyperspectral point-of-measurements (HS-POM) were collected *in vivo* from the adaxial side of healthy and diseased leaflets of the nine tomato plants in the study, in a dark room. For each plant, spectral assessments were performed randomly on nine points of different leaflets, belonging to the 4^th^, 5^th^, and 6^th^ expanded leaflets.

Hyperspectral data were acquired using a Hamamatsu Photonics K.K. TM Series C11697MB spectrometer, which covers a wavelength range of 200-1100 nm with a spectral resolution of 0.6 nm. A transmission optical fiber bundle (FCR-7UVIR200-2-45-BX, Avantes, Eerbeek, The Netherlands) with a range of 200-2500 nm was used along with a stainless-steel slitted reflection probe that was positioned 0.5 cm above the sample surface to capture the leaflet’s spectral signal and direct it to the spectrometer’s entrance lens. A white LED light was placed underneath the leaflet to provide uniform illumination to its entire abaxial surface. The spectral range of the LED emits light from 390 to 800 nm. Therefore, the LED spectra were used as a reference to the spectral range measured by the spectrophotometer and to check measurement and light emission stability (**Figure 1B**). The hyperspectral data were collected using specialized evaluation software (SpecEvaluationUSB2.exe, Hamamatsu Photonics K.K., Japan).

#### 2.2.2 Preprocessing hyperspectral data

**Figure 2:**
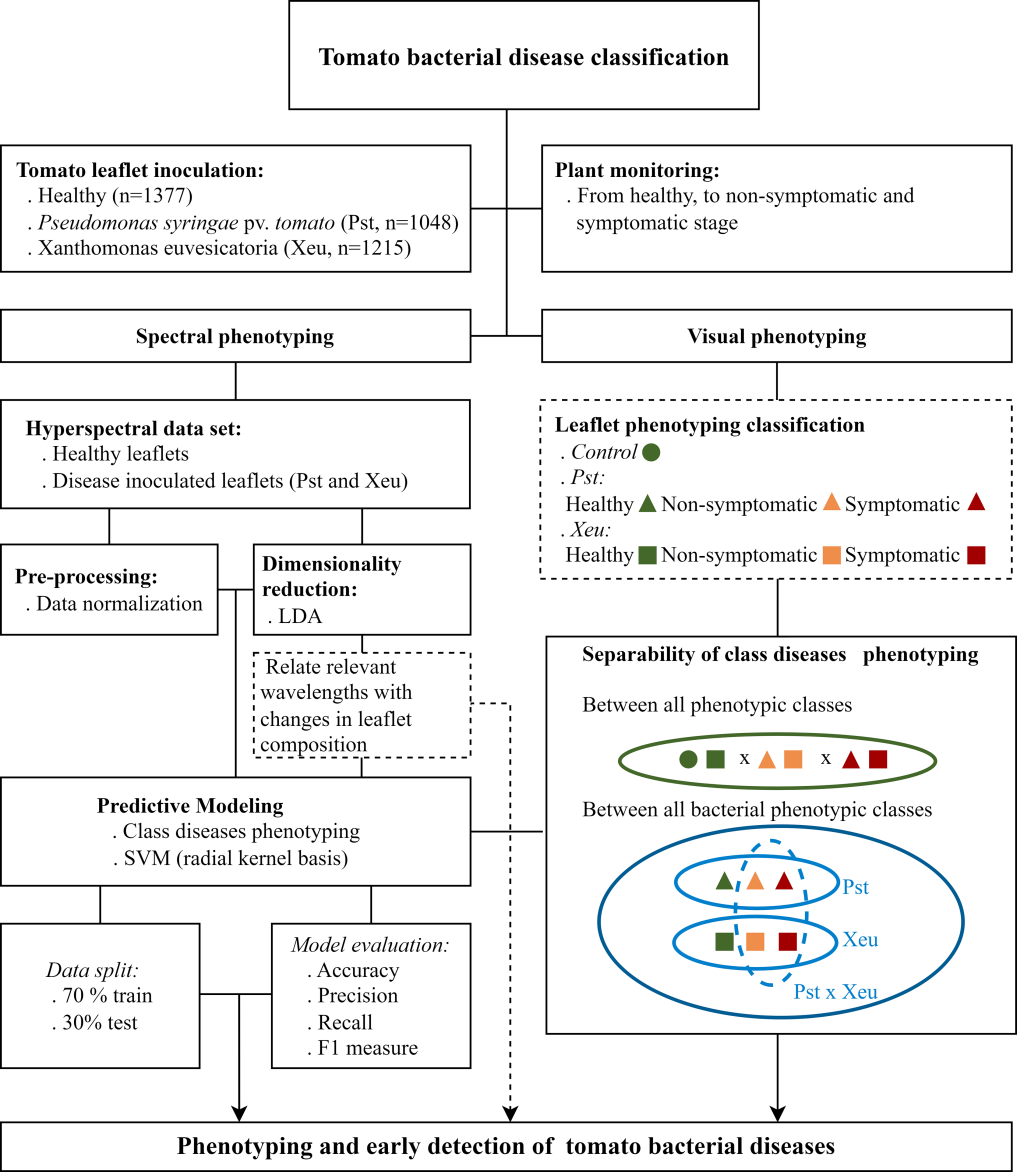
Conceptual diagram for the applied predictive modeling approaches of bacterial tomato leaflet disease.

The performance of the modeling approach in detecting bacterial diseases in tomato leaflets was assessed using only the spectral region of 400 to 800 nm, approximately. This decision was based on the spectral wavelength range of the light LED source used (where possible useful information could be retrieved) and due to the observation of spectral noise near the limits of the equipment’s spectral range, which could negatively affect the performance of the classification process. Therefore, a total of 944 features (wavelength) were used in the development of the prediction modeling (**Figure 2**).

**Figure 3:**
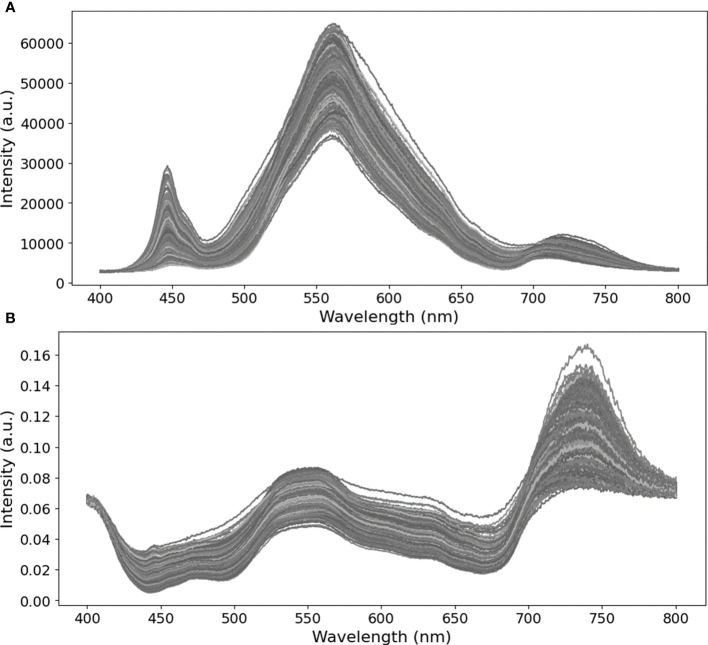
Original (raw, **A**) and normalized **(B)** hyperspectral signatures assessed in tomato leaflets during the experimental assay.


Preprocessing data was performed following spectra normalization (**Figures 2**, **3**). This approach aimed to standardize the data to a common scale, enabling meaningful comparison and analysis across different scenes or datasets. Additionally, it aims to decrease spectral signal oscillations, related to measurement equipment specifications (including devices’ internal noise), variations in data assessment conditions (comprising differences in global spectral trend, total energy, high-frequency noise, and/or local background) (Randolph, 2006), associated to changes in environmental conditions or induced by the operator in the moment of assessment (e.g. variations in sample-sensor distance, uneven illumination conditions, choice of leaflets sample point location, appropriate scan parameters, spectral calibration, among others). This results in model abilities improvement by aiding in class separation (Randolph, 2006; Guezenoc et al., 2019). Furthermore, this process enables the elimination of the spectral response of both the sensor and light source, making possible the transfer of the acquired classifier to a different sensing device. Spectral data retrieved from measurements in tomato leaflets $S{\left(\lambda_{n}\right)}_{m}^{raw}$ were normalized through their division by the white LED source spectral signature $S{\left(\lambda_{n}\right)}^{reference}$ (considering the time of exposure of the spectral measurements), through the computation of the following forming (Equation 1):

(1) $$S{\left(\lambda_{n}\right)}_{m}^{normalized}=S{\left(\lambda_{n}\right)}_{m}^{raw}/S{\left(\lambda_{n}\right)}^{reference}$$

### 2.3 Modeling leaflets symptomatology over time

#### 2.3.1 Data set structure

Seeking bacterial tomato disease classification, spectral signatures from leaflets were then grouped to perform an applied predictive modeling approach related to the plants’ experimental condition. Leaflets were classified according to the plant treatment group and their health status, including the classes: i) healthy, including all the measurements which were performed before bacteria inoculation, and the remaining assessments that were made in non-inoculated plants considered as control plants; ii) non-symptomatic Pst; iii) non-symptomatic Xeu; iv) symptomatic Pst; and v) symptomatic Xeu. All the spectral data collected from tomato leaflets on different dates were unified in a single classification model (**Figures 1**, **2**).

Data classification was, thus, performed seeking the unraveling of spectral phenotyping differences between i) healthy and non-symptomatic diseased tissues (early diagnosis), ii) healthy and diseased tissues (showing visual modifications due to changes in chemical and structural composition), ii) healthy and diseased tissues affected by different bacterial etiological agents (which present distinct host-pathogen specific interactions), iii) and diseased tissues infected by different bacteria species (responsible for causing similar visual symptoms but showing different pathogenic dynamics).

#### 2.3.2 Dimensionality reduction of spectral data

Multi-scale interference in plants’ tissue promotes superimposition on hyperspectral data, resulting in autocorrelations in their spectral signal at several scales (Martins et al., 2022). To mitigate the effects of high dimensional, redundant information, several methodologies have been cited in the state-of-the-art, including dimensionality reduction (DR) approaches (Lapajne et al., 2022; Reis-Pereira et al., 2022). DR techniques are a class of predictor transformations. They can reduce data by creating a minor set of predictors that aim to retain most of the information contained in the original variables. Usually, these approaches generate new predictors which are functions of the original ones (signal extraction or feature extraction techniques) (Kuhn and Johnson, 2013).

This study examined a DR approach called Linear Discriminant Analysis (LDA), generally computed as a pre-processing. It is a supervised learning algorithm used for classification tasks. LDA is usually applied as a feature extraction technique, performed to reduce the dimensionality of the data while maximizing the class separability. It projects the high-dimensional data onto a lower-dimensional space while preserving the discriminative information between classes. In brief, data is projected onto a linear subspace that maximizes the ratio of between-class variance to within-class variance. Thus, the projected data points are as far apart as possible in the new space, while the points belonging to the same class are as close as possible. Therefore, LDA contributes to reducing the problem’s computational complexity and avoiding overfitting. It can also be useful for visualizing the data in a lower-dimensional space, helping interpret patterns in data (Tharwat et al., 2017). Furthermore, this technique was applied since our dataset is not linearly separable, and LDA can organize it in another space with the maximum possible linear separability (Sachin, 2015).

LDA feature space loadings (also called coefficients or weights) were additionally used to infer the most relevant wavelength variables, through the computation of the interquartile range (IQR) for the weights. A threshold at 1.5 times the IQR beyond the upper quartile was established. This process aimed to increase sensitivity to the weight distribution, enabling the capture of outliers and extreme values. An applied predictive classification model was later computed to help deal with the non-linearity of the data.

#### 2.3.3 Machine learning classification model

A Support Vector Machines (SVMs) algorithm was chosen to integrate this modeling strategy. This supervised machine learning algorithm performs classification based on the concept of optimal separating hyperplane (Vapnik, 1999; Mosavi et al., 2021). SVMs are nonlinear approaches that discover the most extensive margin between two classes in feature space. These approaches aim to decrease the error test and model complexity (Ballabio and Sterlacchini, 2012). SVMs can present distinct hyperparameters and kernel forms, which convert raw data inputs from the original user space into kernel space through a user-defined feature map (Patle and Chouhan, 2013; Ding et al., 2021). This study used a radial basis function (RBF) kernel was used since it allows SVMs to capture non-linear relationships between input features and target variables. It may also allocate distinct weights to different points since they learn the decision surface according to the relative importance of the data points in the training set (being well-suited for handling outliers and noisy data) (Xulei et al., 2005). Moredetailed information about the SVM algorithm, including relevant principles and calculation formulas, can be found in Ballabio and Sterlacchini (2012) and in Chang and Lin (2011). The parameters of the SVM applied corresponded to the default values of the algorithm implemented in the ‘Scikit-learn’ machine learning library (Pedregosa et al., 2011), which also can be found in **Table 1**.

**Table 1:** Default parameters of the SVM algorithm of ‘Scikit-learn’ library used in this study.

Parameter	Value	Parameter	Value	Parameter	Value	Parameter	Value
*C*	1.0	*Probability*	False	*Verbose*	False	*Break ties*	False
*Kernel*	rbf	*Shrinking*	True	*Cache size*	200	*Tolerance*	1e-3
*Gamma*	‘scale’ 1/(n_features *X.var())	*Class weight*	None	*Decision function shape*	One-vs-rest (ovr)	*Random state*	None
		*Max iteration*	-1				

0The datasets were divided into training data (70% of random observations) and validation data (30% of the remaining observations) (Kuhn and Johnson, 2013), following a holdout method (Lantz, 2019). The training and validation sets combined the pairs of concurrent measurements of the group and health status and the corresponding values of the predicting variables. A resampling strategy was performed as stated in Reis-Pereira et al. (2022) to reduce the possibility of overfitting (Berrar, 2019; Valier, 2020).

#### 2.3.4 Model performance evaluation

Different metrics were additionally retrieved to investigate model performance, namely the Confusion Matrix (CM), accuracy score (Equation 2), and F1-Score (Equation 3) whose description is detailed in Reis-Pereira et al. (2022). Furthermore, precision (the fraction of correct positive predictions out of all positive predictions, Equation 4) and recall (fraction of correct positive predictions out of all observed positive samples, Equation 5) were also computed using the following formula, where true positive, false positive, false negative, and true negative values are denoted by TP, FP, FN, and TN, respectively:


(2) $$\text{Accuracy}=\frac{T_{P}+T_{N}}{T_{P}+T_{N}+F_{N}+F_{P}}$$

(3) $$\text{F}1\;\text{score}=\frac{2*T_{P}}{2*T_{P}+F_{P}+F_{N}}$$

(4) $$\text{Precision}=\frac{T_{P}}{T_{P}+F_{P}}$$

(5) $$\text{Recall}=\frac{T_{P}}{T_{P}+F_{N}}$$

All the computational analyses were performed in the Jupyter Notebook software using the libraries ‘Matplotlib’ (Ari and Ustazhanov, 2014), ‘numpy’, ‘pandas’ (Betancourt et al., 2019), and ‘Scikit-learn’ (Pedregosa et al., 2011).

## 3 Results

### 3.1 Observational-based phenotyping of leaflets symptomatology over time

#### 3.1.1 PCR validation

Tomato plants were inoculated with Pst and Xeu bacteria, respectively. After spectral analysis, leaf samples from each treatment were tested for the presence of these bacteria. Proper controls from samples known to be positive and negative for Pst and Xeu bacteria were included to confirm the assay results. After the colony PCR reaction, the amplified products were separated by agarose gel electrophoresis and visualized under UV light. The PCR results showed bacteria-specific bands for each bacteria species, namely a 200-base pair (bp) fragment of Pst, and a 713 bp fragment for Xeu, indicating that Pst and Xeu bacteria were present in each inoculation treatment group. No PCR amplification was observed from samples collected from healthy leaves.

#### 3.1.2 Visual and hyperspectral phenotyping

**Figure 4:**
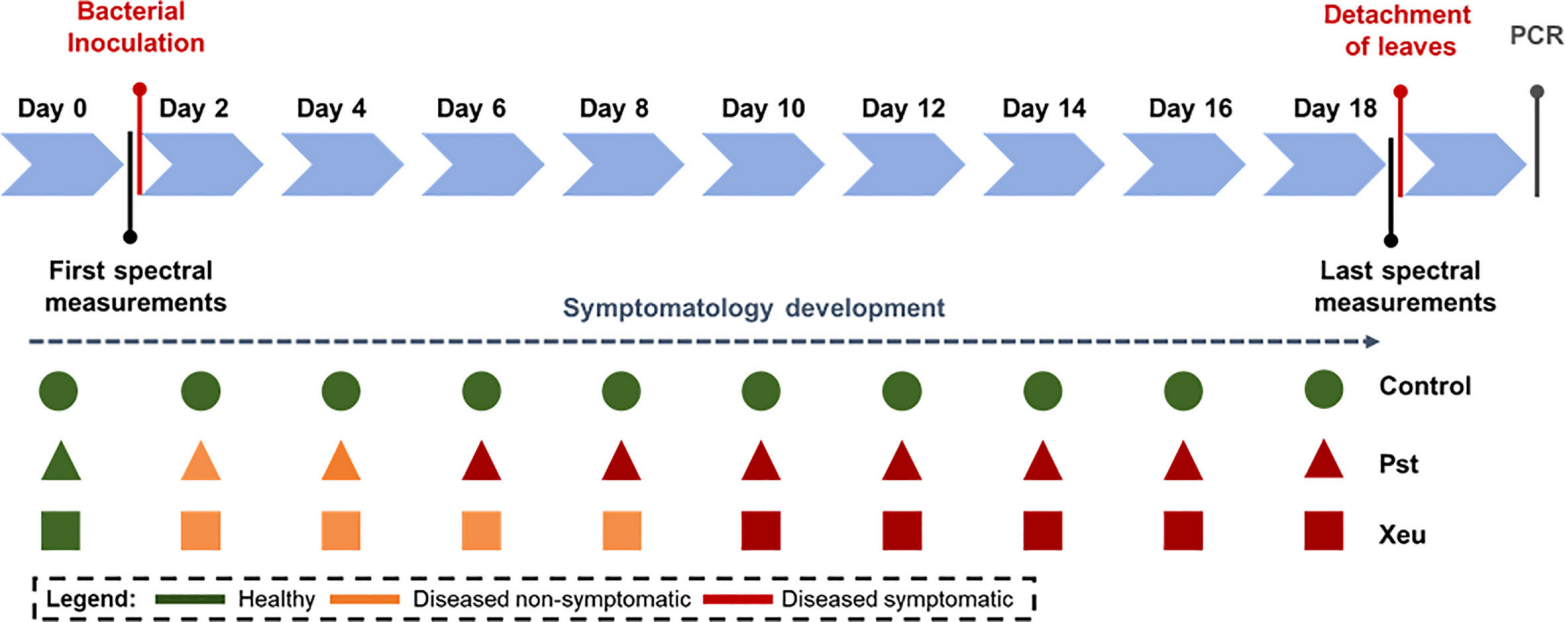
Observational-based phenotyping of leaflet symptomatology over time. Spectral measurements were performed before bacteria inoculation (Day 0), until day 15 (*Pseudomonas syringae* pv. *tomato* diseased leaflets), and 18 days after infection (Control and *Xanthomonas euvesicatoria* diseased leaflets). In the last measurement date, tomato leaflets were detached from each diseased plant and isolated in different bags for later performing the bacteria isolation assay.

Tomato plants infected with Pst bacteria showed the first visual typical chlorotic symptoms mostly between four and five days after infection (DAI). These spots evolved into necrotic lesions at six to seven DAI. In turn, chlorotic lesions in samples inoculated with Xeu mainly developed among twelve to fifteen DAI, only evolving to the necrotic stage at seventeen to eighteen DAI. Pst-infected plants died 12 DAI (**Figure 4**).

**Table 2** presents the dataset structure used, composed of 3478 spectral point measurements, from which 1377 (39.6%) observations correspond to the healthy class. Of these, 1215 (34.9%) assessments belonged to Control leaflets, 81 to measurements performed on Pst leaflets before bacteria inoculation, and 81 to captures made on Xeu leaflets also before bacterial infection. Spectral records performed before symptom appearance reached the value of 844 (24.3%), where 101 (2.9%) measurements belonged to non-symptomatic leaflets inoculated with Pst, and 743 (21.4%) to leaflets inoculated with Xeu bacteria. Lastly, after symptom development, 1257 (36.1%) spectra were captured (866 – 24.90% – from symptomatic Pst leaflets, and 391 – 11.24% – from Xeu symptomatic tissue). Class imbalance is observed due to the disease infection process’s dynamic, resulting in symptoms appearing throughout the measurements dates at different rates (**Table 1**). Spectral assessments were performed during 18 DAI for Control and Xeu leaflets. For Pst, the process was only made until 15 DAI because the plants presented high-stress levels, and leaf dehydration after this date, interfering with the spectral signal recording (**Figure 1**; **Table 1**).

**Table 2:** Spectral data characterization of the measurements randomly performed on tomato leaflets (healthy, diseased with *Pseudomonas syringae* pv. *tomato* – Pst –, and diseased with *Xanthomonas euvesicatoria* – Xeu), showing the number of assessments made by class and date.

Days after Infection (DAI)	Non-inoculated	Inoculated classes			
		Non-symptomatic		Symptomatic	
		Xeu	Pst	Xeu	Pst
0	243*	0	0	0	0
3	81	81	81	0	0
4	81	81	17	0	64
5	81	81	3	0	78
6	81	81	0	0	81
7	81	81	0	0	81
8	81	81	0	0	81
10	81	71	0	10	80
11	81	63	0	18	80
12	81	33	0	48	80
13	81	28	0	53	81
14	81	28	0	53	79
15	81	34	0	47	81
17	81	0	**–**	81	**–**
18	81	0	**–**	81	**–**
**Total** **(n=3478)**	**1377**	**743**	**101**	**391**	**866**
	39.6%	21.4%	2.9%	11.2%	24.9%

* Including all plants. After day 0, only Control plants belong to this class.

Bold values correspond to the total number of assessments.

**Figure 5:**
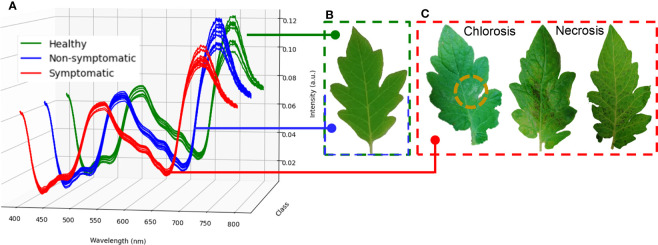
Mean normalized spectra of healthy, non-symptomatic, and symptomatic leaflet measurements for the first ten measurements performed (12 DAI, **A**). Healthy and non-symptomatic infected leaflets presented equal visual phenotype **(B)**. With infection evolution over time, chlorotic symptoms started to appear and later turned into necrotic lesions **(C)**.

**Figure 6:**
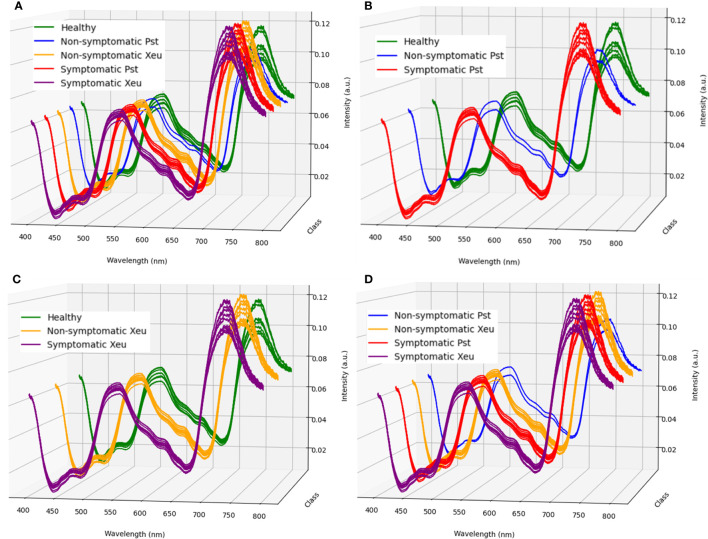
Mean normalized spectra for all classes in study (i.e., healthy, non-symptomatic, and symptomatic *Pseudomonas syringae* pv. tomato – Pst – leaflet measurements, and non-symptomatic *Xanthomonas euvesicatoria* – Xeu – assessments) for the first ten measurements performed (12 DAI, **A**). Different behaviours of healthy samples compared to Pst **(B)**, and Xeu **(C)** diseased leaflets are shown, as well as, between non-symptomatic and symptomatic diseased leaflets **(D)**.

Hyperspectral signatures captured in healthy leaflets showed the typical spectral behavior of healthy green tissues. On the other hand, spectral assessments belonging to disease leaflets (both with Pst and Xeu bacteria) presented deviations in their format (**Figure 5**). Thus, a more detailed analysis was performed for these measurements to evaluate the spectral modifications caused by the different bacteria, resulting in a higher number of classes in the study. Spectra signatures belonging to Pst inoculated samples had a more distinct spectral curve (for both, non-symptomatic and symptomatic stages) compared to the healthy measurements, showing higher intensity on the wavelength ranges of approximately 430 to 520 nm, and 560 to 680 nm. Nevertheless, the lower intensity was captured from 710 to 800 nm (**Figures 6A, B**). Xeu-inoculated tissues also displayed modification in their spectral signature in these regions. The intensity measured in the first two spectral intervals was marginally higher than the one captured on healthy leaflets. However, a more evident variance was observed in the 710 to 780 nm range (**Figures 6A, C**). When measurements belonging to disease samples were compared, the data showed differences between the samples infected with the different etiological agents. Pst measurements (for both non- and symptomatic stages) demonstrated greater intensity in the ranges of 430 to 520 nm, and 560 to 680 nm, but lower intensity in the 710 to 800 nm interval (**Figures 6A, D**).

### 3.2 Hyperspectral sensing-based phenotyping of leaflets symptomatology over time

#### 3.2.1 Reducing the spectral dataset dimensionality

**Figure 7:**
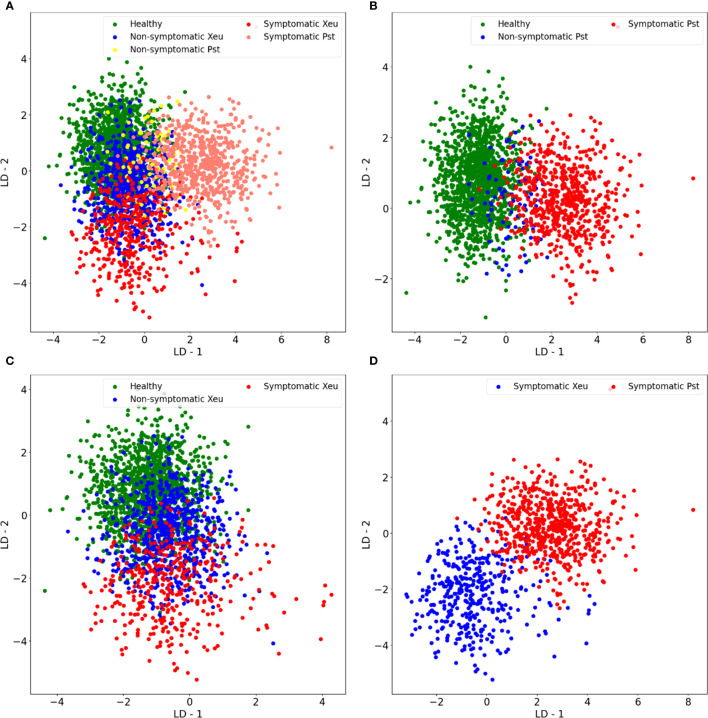
Scatter plots of the outcomes of the application of Linear Discriminant Analysis on the normalized data, for Linear Discriminant 1 (LD1) and Linear Discriminant 2 (LD2), when were used all the classes in study **(A)**, only healthy and Pseudomonas syringae pv. tomato infected samples **(B)**, just healthy and *Xanthomonas euvesicatoria* diseased leaflets **(C)**, and only diseased symptomatic samples **(D)**.

A Linear Discriminant Analysis (LDA) was performed to reduce the dimensionality of the normalized dataset, organizing the spectral observations in a new space as the maximum linear separability possible. LDA results were plotted and showed spectral separability between the different classes studied (**Figure 7A**). It was possible to see an evolution pattern through LD 1 for spectral data belonging to healthy, and Pst diseased leaflets regardless of whether they exhibit symptoms or not (**Figures 7A, B**). In turn, healthy and Xeu-diseased leaflets (including, non- and symptomatic data) presented a spectral separation gradient through LD2 (**Figures 7A, C**). When data of diseased leaflets infected with distinct bacteria were compared, it was possible to observe a divergence gradient between the LD1 and LD2, especially at the symptomatic stage (**Figures 7A, D**). Since data presented a non-linear characteristic, overlapping between classes was observed. Thus, these findings demonstrated the efficacy of the LDA technique for reducing the dataset dimensionality to the most important features. LDA’s DR results were, then, applied in the following steps of the modeling process helping in the classification task and avoiding overfitting.

**Figure 8:**
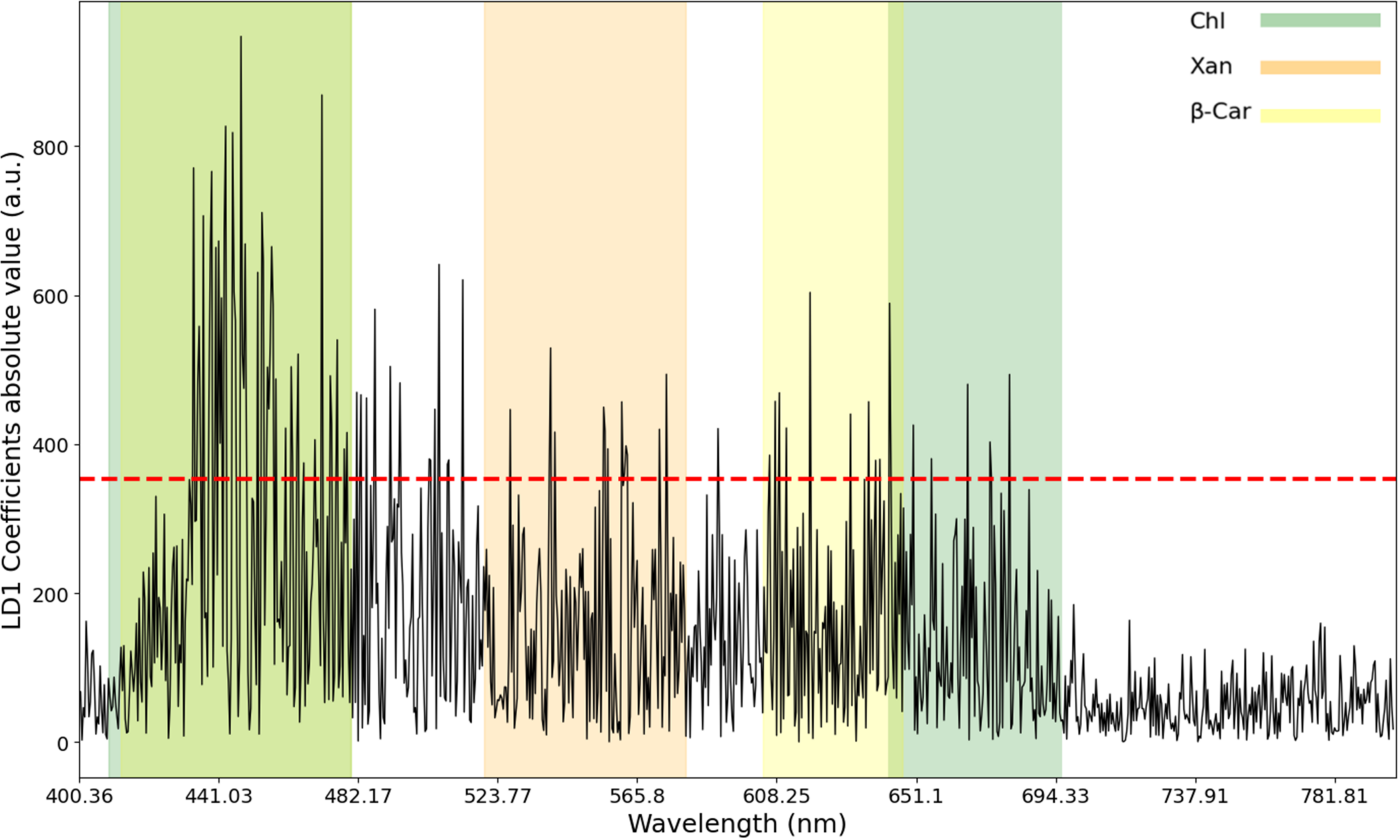
Absolute values of the coefficients results of Linear Discriminant Analysis for Linear Discriminant 1. Forty-four spectral wavelengths were identified as relevant when variable weights were computed. These variables coincided with the absorption spectra of different photosynthetic pigments, namely chlorophylls (Chl, highlighted in green for chlorophyll), and carotenoids (β-carotenes, β-car, highlighted in yellow; and xanthophyll’s, Xan, highlighted in orange).

The most relevant wavelength variables for LD1 were assessed based on their coefficients, equaling 44 features. These variables were mostly located in the blue-green and red VIS regions of the electromagnetic spectrum (blue - 434.9, 435.72, 438.17, 438.58, 440.21, 441.44, 442.67, 443.08, 445.53, 445.94, 448.4, 448.81, 494.6 nm; green - 503.74, 508.74, 527.53 nm; red - 556.09, 562.0, 562.84, 590.37, 607.82, 609.1, 611.24, 618.5, 643.36, 650.24, 673.97, 680.02 nm), coinciding with the wavelength absorption range of chlorophylls (430 to 480 nm, and 640 to 700 nm), and carotenoids pigments, namely β-carotenes (whose primary and secondary absorption peaks are respectively located at 450 to 480 nm, and 600 to 650), and xanthophylls (520 to 580 nm) (**Figure 8**). This coincides with the action of Pst and Xeu bacteria on tomato leaves’ levels of photosynthetic pigments during the infection process.

Other plants whose metabolites are affected by these two bacteria also have their absorption spectrum coinciding with the selected wavelengths of LD1, namely some phenolic compounds (e.g., flavonoids, 400 to 500 nm), and composts derived from chlorophylls decomposition, namely pheophytins (400 to 500 nm, and 600 to 700 nm) (**Figure 8**).

Applied predictive classification modeling was, then, performed using the LDA-reduced normalized data (including all classes: i) healthy; ii) non-symptomatic diseased Pst leaflets; iii) non-symptomatic Xeu samples; iv) symptomatic inoculated Pst tissues; v) symptomatic Xeu observations) and an SVM algorithm with a Radial Basis Function (RBF) kernel. The model was trained using 70% (2434) of the spectral observations (randomly selected), and then, it was validated using the remaining 30% (1044) of the observations (test set), and the complete dataset. The test set comprised 413 healthy samples, 30 non-symptomatic Pst disease leaflets, 223 non-symptomatic Xeu, 260 symptomatic Pst observations, and 118 symptomatic Xeu.

**Figure 9:**
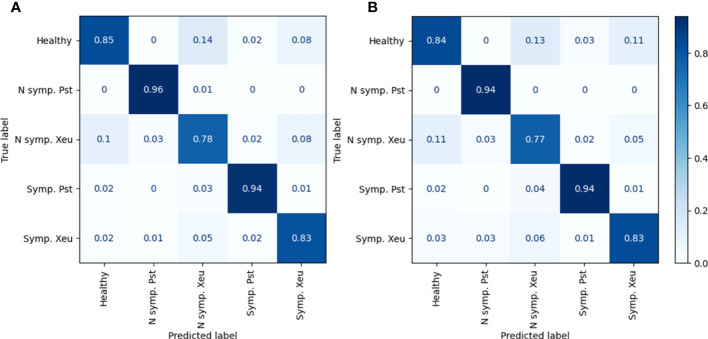
Confusion Matrix of the percentage of predicted samples for each class (column) that were correctly classified for each true class (row), for the complete **(A)** and test **(B)** sets. (N Symp., Non-symptomatic; Sym., Symptomatic).

The developed model performed well for both the test set and the complete dataset. The model achieved an accuracy of 0.85 for the test set and 0.86 for the complete dataset, indicating that it can correctly classify most of the measurements (**Table 3**; **Figure 9**). Furthermore, it demonstrated high metric values (precision, recall, and F1-score) for all the classes, indicating that it can identify both healthy and infected measurements. In detail, higher precision, recall, and F1-score values were found for the healthy and non-symptomatic Pst leaflets measurements (**Table 3**). This shows that the model more easily predicted spectral assessments belonging to these classes. Nevertheless, it showed more difficulties in classifying measurements of Xeu inoculated leaflets, especially those captured before symptom appearance (indicated by lower metric scores). It is important to note that the model’s performance was consistent across both the test set and the complete dataset, indicating that the model is robust and can be used to classify new spectral samples accurately.

**Table 3:** Performance metrics for the classification SVMs-based model using all the data (train and test set – All), only the train set (Trn) and only the test set (Test).

Class of leaflets status	Precision			Recall			F1-score			Accuracy		
	*Trn*	*All*	*Test*	*Trn*	*All*	*Test*	*Trn*	*All*	*Test*	*Trn*	*All*	*Test*
*Healthy*	0.86	0.85	0.84	0.89	0.89	0.88	0.88	0.87	0.86	0.89	0.85	0.88
*N Sym. Pst*	0.97	0.96	0.94	0.86	0.90	1.00	0.91	0.93	0.97	0.86	0.90	1.00
*N Sym. Xeu*	0.78	0.78	0.77	0.74	0.74	0.74	0.76	0.76	0.75	0.74	0.74	0.74
*Sym. Pst*	0.94	0.94	0.94	0.95	0.94	0.93	0.95	0.94	0.93	0.95	0.94	0.93
*Sym. Xeu*	0.83	0.83	0.83	0.80	0.79	0.77	0.81	0.81	0.80	0.79	0.79	0.77
Weighted Avg ± s.d	0.86± 0.07	0.86 ± 0.07	0.85± 0.07	0.86± 0.07	0.86 ± 0.08	0.85± 0.10	0.86± 0.07	0.86 ± 0.07	0.85 ± 0.08	0.86 ± 0.07	0.86 ± 0.08	0.85 ± 0.10

SVMs, Support Vector Machines; Trn, Train; N Symp., Non- symptomatic; Sym., Symptomatic; Avg., Average; s.d., standard deviation.

Model predictions for the non-symptomatic Pst class did not present any misclassification in the test set. In the complete dataset, the model accurately predicted 96% of the spectral measurements but missed 1% of the predictions, which it classified as assessments made on non-symptomatic leaflets infected by Xeu (**Figure 9**). Symptomatic spectral captures performed in Pst diseased leaflets were correctly categorized in 94% of the cases for both the test and complete sets. Nevertheless, the model mistakenly classified these assessments as non-symptomatic Xeu observations in 4% and 3% of the cases, and as healthy samples in 2% when the test set and complete dataset were used, respectively. Predictions of Xeu spectral assessments were more challenging to the model, presenting a higher number of wrong classifications in the non-symptomatic class than in the remaining classes studied. In fact, the model successfully classified 77% of the measurements of this class in the test set, and 78% when all data was used. However, it attributed 11% and 10% of the measurements as healthy, 5% and 8% as symptomatic diseased Xeu leaflets assessments, 3% as non-symptomatic inoculated Pst observations, and 2% as symptomatic Pst captures, when the test set and complete dataset were used, respectively. The model showed more efficacy in identifying symptomatic Xeu leaflets measurements, predicting 83% of these samples in the test and complete datasets. In terms of missed classifications, it predicted 6% and 5% of the assessments as non-symptomatic, 3% and 2% as healthy, 3% and 1% as non-symptomatic spectral captures of Pst infected leaflets, and 1% and 2% as symptomatic Pst, in the test set and complete dataset, respectively (**Figures 9A, B**).

**Figure 10:**
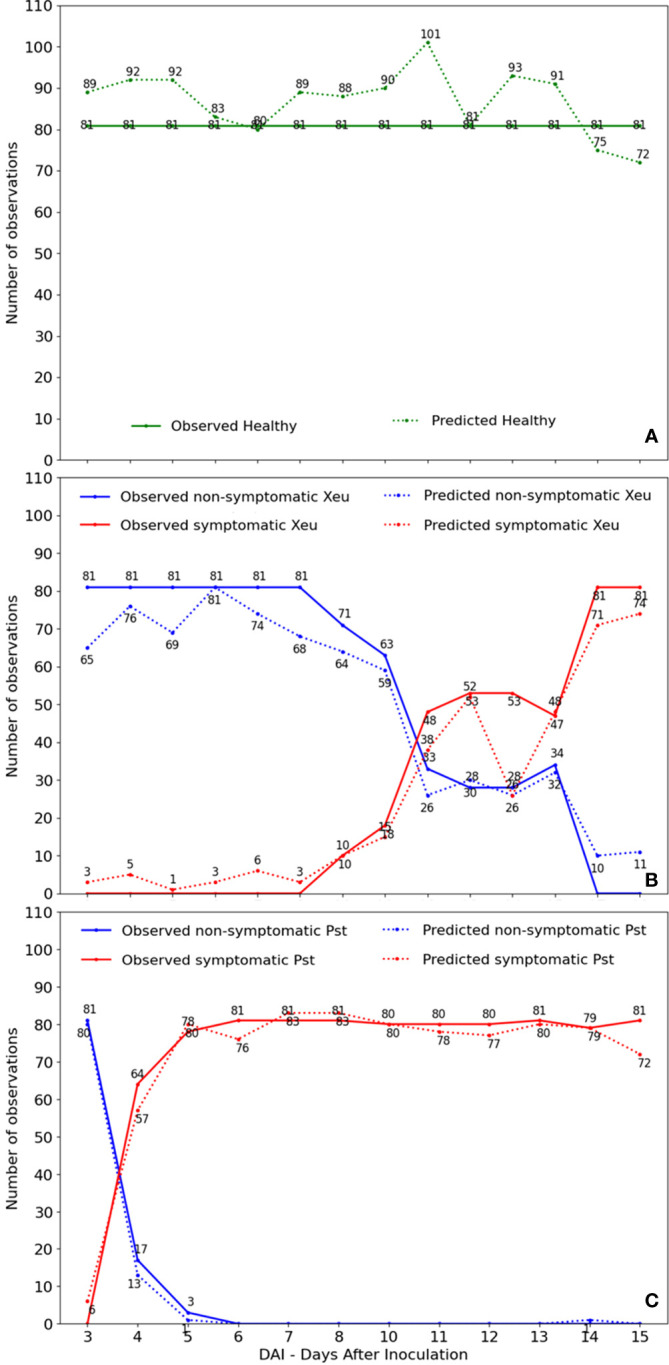
Number of observed and predicted samples by date of measurement for healthy **(A)**, *Xanthomonas euvesicatoria* diseased **(B)**, and *Pseudomonas syringae* pv. *tomato* diseased **(C)** leaflets’ assessments.

For the complete dataset prediction, we investigated the number of misclassifications per class and date (**Figure 10**). As expected, the observed tendency for healthy spectral assessments showed a regular number of observations per date (81). Nonetheless, the developed model categorized more samples than the true value per date, except for 7, 13, 17, and 18 DAI. On the other hand, the spectral model consistently underfit the infected Xeu leaflets, regardless of whether they exhibit symptoms or not (**Figure 10A**).

In plants inoculated with Xeu, discrepancies between observed and predicted classes are more evident in the non-symptomatic Xeu class in the observations recorded up to 10 days after infection. During this period, which included seven measurement dates of the non-symptomatic Xeu class, 53 observations were recorded below the predicted value of the developed model. In contrast, the healthy class accumulated 47 observations above the predicted value during the same period. Furthermore, according to the confusion matrix results (All data), 10% (148) of the non-symptomatic Xeu observations were misclassified as healthy. Considering the period up to 10 days after infection (data not shown), out of the 150 observations wrongly classified as healthy, 100 were from the non-symptomatic Xeu class. These results indicate that in the early stages of Xeu-induced disease infection, the symptoms developed in the plant leaflets are not strong enough for the developed model to distinguish them from healthy observations efficiently. Therefore, the non-symptomatic Xeu class, compared to other tested classes, exhibits the lowest model performance indicators (all data: accuracy 0.74, precision 0.78, recall 0.74, and F1-score 0.76). For the non-symptomatic stage, the actual observations presented a stable pattern until 8 DAI, and after a sharp drop was observed until 13 DAI, where the rate of infected leaflets increased up to 65%. A stable number of observations was maintained until 15 DAI, after which a period of exponential increase in observed symptomatic spectral measurements was registered. After this day, all leaflets were symptomatic. The model was rigorous in discriminating non-symptomatic Xeu leaflet measurements after 9/10 DAI, presenting a percentage of error inferior to 10% (correctly classifying 64 of the 71 observations) when about 90% of the sampled leaflets (71 of the initial 81 assessments) still didn’t show any typical symptoms of the disease (**Figure 10B**).

For the prediction of the Pst disease samples, the non-symptomatic phase was very similar for both observed and predicted. Nevertheless, the prediction of the symptomatic phase showed irregularities between the five and seven days (corresponding to the dates were necrosis appeared). Is possible to observe that most of the Pst inoculated leaflets (79%) started to show the first symptoms of the disease 4 DAI. The number of symptomatic sampled leaflets increased until 6 DAI, where all the leaflets assessed were symptomatic (**Figure 10C**).

## 4 Discussion

Plant infectious diseases are critical in agriculture and food security, impacting crop yields and quality. Understanding and effectively managing them is crucial for more sustainable agriculture, based on more preventive measures and early diagnosis.

The suitability of spectral phenotyping based on hyperspectral spectroscopy point-of-measurement (HS-POM) for diagnosing bacterial infectious diseases in tomato plants, namely bacterial speck and spot, was evaluated. In this approach, light penetrates the leaflet tissue and undergoes internal reflections, before ultimately being redirected to the spectrometer *via* a central fiber optics pinhole. This method ensures that all light reaching the sensor interacts with the leaf tissues, thereby maximizing the spectral information from all internal tissues, including any changes caused by the interaction between the host and bacteria.

An applied predictive model integrating an SVM algorithm showed the capacity to accurately classify healthy and diseased tomato leaflets at various stages of disease development (specifically healthy, non-symptomatic diseased Pst, non-symptomatic disease Xeu, symptomatic Pst, and symptomatic Xeu). Even before symptom appearance, it showed a classification accuracy of 74% for Xeu and 100% for Pst diseased leaflets measurements, and a weighted average accuracy, precision, recall, and F1-score of 85%.This model was, thus, capable of categorizing healthy, disease (both non-symptomatic and symptomatic), and disease leaflet tissues infected with distinct bacteria species (both before and after symptom appearance), being coherent with visual phenotyping and PCR results. These outcomes, thus, demonstrate the suitability of this technique for performing an early disease assessment and class distinction (according to the phytosanitary health status, and type of pathogen responsible for the infection). This is extremely valuable since crops in the field are generally exposed to variable environmental and phytosanitary conditions and vulnerable to different types of abiotic and biotic stresses (which may cause similar visual lesions, difficult to distinguish by the naked eye). Also, bacterial spot and speck of tomato can develop in 6 to 14 days, depending on several factors (e.g., environmental conditions, pathogen strain, infection severity, inoculum concentration, and the susceptibility of the plants’ variety) (Horst, 2013; Borkar and Yumlembam, 2016), and their spread among several plants in a production field is not immediate and may take time to occur. Thus, early diagnosis is crucial to prevent disease spread, promote preventive treatments, and lead to environmentally friendly practices, promoting precision agriculture principles.

LDA computation revealed spectral divergence between the different classes in study through LD1 and LD2 and uncovered relevant wavelengths for diagnosing the diseases caused by *Pseudomonas syringae* pv. *tomato* (Pst), and *Xanthomonas euvesicatoria* (Xeu). These were mostly located in the blue-green and red visible regions of the electromagnetic spectrum, corresponding to chlorophyll (mainly: 430 to 480 nm, and 640 to 700 nm) and carotenoid pigments’ absorption spectra (i.e., 450 to 480 nm, 520 to 580 nm, and 600 to 650 nm), indicating modifications in the photosynthetic pigment’s levels throughout the infection process. These findings are aligned with the impact of both bacteria species on host leaves’ pigments values during infection, which start prior to symptoms appearance and became more pronounced with the formation of chlorotic and necrotic lesions. In this medium/late stages of infection, the breakdown of chlorophyll, in particular, can result in a subsequent accumulation of pheophytins (brown pigments, whose maximum absorption peak is located at 660-670 nm, and secondary peak around 430-450 nm), which also affect plant spectral behavior (Bhandari et al., 2015). Also, spectral divergences in the 700 to 800 nm range may indicate that structural components of leaves are affected during the infection process, resulting in the degradation of leaf structures along disease development. Spectral divergence between diseased leaves infected by different bacteria may be related to the production of specific molecules by each pathogen, which may affect the host spectral signature. As an example, Pst produces a phytotoxin called coronatine which alters chlorophyll fluorescence (by modifying the photosystem II – PSII) and can affect the absorption and scattering of light by plant tissues, leading to modifications in the spectra (Zhang et al., 2021). In turn, the host plant can activate different defense responses when in contact with distinct pathogens, triggering a series of biochemical and molecular responses, which also promote spectral modifications in the visible wavelength ranges. An example are phytoalexins (e.g., flavonoids), whose production was hypothesized to be related to increased plants’ spectral reflectance in the VIS range (Leucker et al., 2016).

Hence, the present research findings demonstrate that HS-POM holds promise as an effective, fast, and cost-effective overtime method for early diagnosis of two bacterial infections caused by distinct pathogen species *in vivo* tomato plants, and for unraveling specific host-pathogen spectral dynamics. In the future, it is advisable to conduct further analysis, entailing the expansion of the dataset under study, test various values for SVM algorithm parameters, and enhance the modeling algorithms, among other potential approaches. This study corroborates previous research performed by our team using HS-POM for the early detection of bacterial tomato spot caused by Xeu bacteria. The spectral response properties of disease tomato leaves presented a divergent behavior when compared to healthy tissues, even before symptom appearance. This tendency was more evident in the absorption regions of photosynthetic pigments (namely, chlorophyll). A Principal Component Analysis (PCA) allowed the identification of relevant discriminative wavelengths at approximately 454-654 nm (Reis-Pereira et al., 2021), coinciding with the wavelengths identified by the LDA approach.

Other studies also demonstrated the potential of hyperspectral data and SVM-based classification modeling for disease diagnosis, presenting similar model evaluation metrics. As an example, Cen et al. (2022) studied the possibility of early detection of bacterial wilt in tomato by applying a portable hyperspectral spectrometer. Their model combined Genetic Algorithms and SVM and achieved overall accuracies (OA) of 90.7% in the distinction of healthy and symptomatic tissues. Tomaszewski et al. (2023) also demonstrated the suitability of hyperspectral measurements and machine learning for the early detection of anthracnose, bacterial speck, early blight, late blight, and septoria leaf, using a temporally-aggregated approach. When all the data were analyzed, the researchers found that the best-quality classification approach (integrating a Ridge classifier) presented an F1 score ranging from 0.71 to 0.95 (0.84 average) for the period of the first two weeks from inoculation. Despite being possible to find research diagnosing different types of biotic stress agents in the same assay, they are usually more related to fungi identification. Scarce results can be retrieved for studies comparing the assessment of diseases caused by different types of bacterial species.

Besides tomato crop studies, hyperspectral measurements were also valuable to achieve disease diagnosis in several plant species with agronomic interest. For instance, Rumpf et al. (2010) studied the suitability of hyperspectral reflectance, SVM, and vegetation indexes (VIs) for detect and classify diseases on sugar beet leaves (namely, Cercospora leaf spot, leaf rust, and powdery mildew). Early differentiation between healthy and inoculated plants, as well as among specific diseases was achieved using SVM, registering accuracy values ranging from 65 to 90%. When data belonging to healthy and diseased leaves (including all the samples affected by the three different pathogens) was used, the classification model achieved an accuracy higher than 86%. Furthermore, Tian et al. (2021) also proved the efficacy of spectroscopy and machine learning techniques for rice leaf blast infection from non-symptomatic to mild stages. An approach integrating an SVM algorithm achieved maximum classification accuracies of over 80% and 83% for the early infection stage of the 2018 and 2019 experiments.

The desirable possibility of applying hyperspectral data for in-field detection and classification of diseases was also proved. Deng et al. (2019) also demonstrated the possibility of applying hyperspectral reflectance in-field detection and classification of citrus Huanglongbing disease. They developed an SVM learner which achieved 90.8% accuracy in healthy, asymptomatic, and symptomatic discrimination. Our team, likewise demonstrated the capability of using HS to diagnose *in situ* bacterial canker disease, caused by another *Pseudomonas* pathovar, specifically *Pseudomonas syringae* pv. *actinidiae* (also known as Psa). Asymptomatic and symptomatic leaves were successfully discriminated through the computation of several modeling approaches involving different feature selection techniques, as well as multivariate analysis methods and machine learning algorithms. The best predictive classification model for discriminating the bacterial kiwi canker disease showed an overall accuracy of 0.85, with an F1-score (Reis-Pereira et al., 2022). These findings suggest that hyperspectral data can be successfully used to predict plant diseases both indoor and infield conditions, caused by different etiological agents (e.g., fungi, bacteria, and virus), in both herbaceous and woody crops. Despite these encouraging findings, it is important to highlight that comparison between different research can be challenging due to the pathogens in study (e.g., generally disease detection using HS is mostly performed for fungal infections), host-pathogen specific interactions, number of samples used, number of classes analyzed, moment of disease assessment (before or after symptoms appearance, in a specific date or overtime), environmental and experimental conditions on the moment of data acquisition, among others. Thus, future studies using tomato plants should be performed to evaluate the efficacy of this approach.

In summary, point-of-measurement Hyperspectral Spectroscopy devices combined with applied predictive models seem to be suitable for spectral phenotyping of bacterial-infected tomato leaflets. Nevertheless, HS-POM approaches as plant disease diagnostic methods are still in a very initial phase of development, and their Technology Readiness Levels (TRLs) must be improved. Standardized protocols for hyperspectral data acquisition should be developed aiming to uniformize the diagnosis processes and reduce noise and undesired spectral interferences. Also, more research on different host-pathogen interactions must be performed. Classification models developed under controlled conditions can be highly effective and constitute an important step for improving and maturing the diagnosis process. In fact, these models usually can detect symptoms earlier than in field assays (since optimal conditions for bacteria development, dissemination, and infection can be recreated), making the process faster and specific to the host-pathogen in study. Hence, the more challenging in-field application of HS-POM for disease diagnosis, posing additional complexities due to sensing system configurations (e.g., light source, probe position, among others), can be established and improved.

Future studies must be conducted to complement these gaps and validate the application of this technique as a suitable tool for accurately predicting different host-pathogen interactions and their impact on the crops’ spectral signature. Further methodological developments are necessary to address these challenges and enhance the suitability of HS-POM for real-time disease monitoring and precision agriculture systems. Moreover, the implementation of feature selection techniques and dimensionality reduction approaches can help identify relevant wavelengths for distinguishing crop diseases, making possible the development and production of more cost-effective multiband sensors. These devices can be integrated into different platforms, enabling spectral data acquisition at different levels, such as leaf, single-plant, and canopy scales.

## 5 Conclusion

The present research explored the application of *in-vivo* POM hyperspectral spectroscopy combined with applied predictive modeling to classify bacterial leaf diseases in tomato crop, caused by *Pseudomonas syringea* pv. *tomato* and *Xanthomonas euvesicatoria*. Healthy leaves showed a characteristic spectral signature of green and photosynthetically active vegetation, while symptomatic leaves presented differences in their spectral signature in the VIS region. Spectral differentiation between healthy and diseased leaves was observed, even in the early stages of the infection process, when diseased samples didn’t present any visual symptom (asymptomatic stage). Furthermore, plants inoculated with Pst bacteria also revealed a divergent spectral behavior from the ones infected with Xeu, indicating that this approach may be suitable for differentiating the etiological agents. Colony PCR also validated the effectiveness of the infection process for each sample group. The developed model revealed a classification accuracy for the test set of 100% for Pst disease leaflets without any visual symptom, and of 74% for Xeu disease leaflets also in a non-symptomatic stage of infection. The developed model achieved a weighted average accuracy, precision, recall, and F1-score of 85% for the test set. These findings strength the applicability of applied predictive classification modeling using HS-POM to early detect bacterial crop diseases. Nevertheless, complementary, and additional studies are recommended to unravel the host-pathogen interactions and their impact on the crop spectral signature. More economic, multiband devices can be developed hereafter considering the features selected for crop disease discrimination. Thus, different agronomic tasks (including mapping, monitoring, scouting, and treatment of plant diseases) can be performed more accurately with this methodology, fulfilling the precision agriculture concept. Spectroscopy sensors can also be mounted on diverse platforms, creating different functioning measurement systems, which can assess spectral data on distinct levels (namely, leaf, single-plant, and canopy scale).

## Data availability statement

The original contributions presented in the study are included in the article/supplementary files, further inquiries can be directed to the corresponding author/s.

## Author contributions

MRP, FS, FT, and MC contributed to the conception and design of the study. MRP organized the database. MRP and FS performed the data analysis. MRP and FS performed the applied predictive modeling. MRP wrote the first draft of the manuscript. MRP, FS, FT, and MC wrote sections of the manuscript. MRP and MC developed the figures to be integrated into the manuscript. All authors contributed to the article and approved the submitted version.
